# Effects of Helioxanthin Derivative-Treated Human Dental Pulp Stem Cells on Fracture Healing

**DOI:** 10.3390/ijms21239158

**Published:** 2020-12-01

**Authors:** Daiki Yamakawa, Yoko Kawase-Koga, Yasuyuki Fujii, Yuki Kanno, Marika Sato, Shinsuke Ohba, Yoshiaki Kitaura, Miki Kashiwagi, Daichi Chikazu

**Affiliations:** 1 Department of Oral and Maxillofacial Surgery, Tokyo Medical University, 6-7-1 Nishishinjuku, Shinjuku-ku, Tokyo 160-0023, Japan; daiki19@tokyo-med.ac.jp (D.Y.); yasuyuki.fujii0730@gmail.com (Y.F.); ykanno13@tokyo-med.ac.jp (Y.K.); d06048@yahoo.co.jp (M.S.); chikazu@tokyo-med.ac.jp (D.C.); 2 Department of Oral and Maxillofacial Surgery, Tokyo Women’s Medical University, 8-1 Kawada-cho, Shinjuku-ku, Tokyo 160-0023, Japan; 3 Department of Cell Biology, Institute of Biomedical Sciences, Nagasaki University, 1-7-1 Sakamoto, Nagasaki 852-8588, Japan; s-ohba@nagasaki-u.ac.jp; 4 Department of Bioengineering, School of Engneering, The University of Tokyo, 7-3-1 Hongou, Bunkyo-ku, Tokyo 113-0033, Japan; yoshiakikitaura940@gmail.com; 5 Department of Oral-Maxillofacial Surgery and Orthodontics, University of Tokyo Hospital, 7-3-1 Hongou, Bunkyo-ku, Tokyo 113-0033, Japan; m1k1chiik@gmail.com

**Keywords:** human dental pulp stem cells, helioxanthin derivative, fracture healing, cell sheet, cell transplantation

## Abstract

Bone defects affect patients functionally and psychologically and can decrease quality of life. To resolve these problems, a simple and efficient method of bone regeneration is required. Human dental pulp stem cells (DPSCs) have high proliferative ability and multilineage differentiation potential. In our previous study, we reported a highly efficient method to induce osteogenic differentiation using DPSC sheets treated with a helioxanthin derivative (4-(4-methoxyphenyl)pyrido[40,30:4,5]thieno[2,3-b]pyridine-2-carboxamide (TH)) in a mouse calvarial defect model. However, the localization of the DPSCs after transplantation remains unknown. Therefore, in this study, we investigated the localization of transplanted DPSCs in a mouse fracture model. DPSCs were collected from six healthy patients aged 18–29 years, cultured in normal medium (NM), osteogenic medium (OM), or OM with TH, and fabricated them into cell sheets. To evaluate the efficacy of fracture healing using DPSCs treated with OM+TH, and to clarify the localization of the transplanted DPSC sheets in vivo, we transplanted OM+TH-treated DPSC sheets labeled with PKH26 into mouse tibiae fractures. We demonstrated that transplanted OM+TH-treated DPSCs sheets were localized to the fracture site and facilitated bone formation. These results indicated that transplanted OM+TH-treated DPSCs were localized at fracture sites and directly promoted fracture healing.

## 1. Introduction

Bone defects, such as fractures, chronic periodontitis, tumors, and cleft bone defects, affect patients both functionally and psychologically, and can lead to poor quality of life due to the long time required for fracture healing. The process of fracture healing follows a partially similar process to endochondral ossification, which is the process of long bone development in the embryo [1]. In endochondral ossification, skeletal progenitor cells migrate to the fractured site, and after condensing, they differentiate into chondrocytes and osteoblasts. These cells subsequently form cartilage callus and calcify and replace the bone [2,3,4]. The mechanism of fracture healing is complex. Towards the establishment of efficient fracture treatment approaches, it is important to understand the detailed mechanisms of membranous ossification, endochondral ossification, and bone development.

Currently, autogenous and allogeneic bones as well as artificial materials are used to treat bone defects. However, these treatments have risks of infection and bone resorption as well as ethical issues. To resolve these problems, a simple, safe, and efficient method of transplantation is required.

Recently, cell therapy has attracted attention and many studies are using animal models to analyze the functions of stem cells including pluripotent stem cells, ES cells, and somatic stem cells. Human dental pulp stem cells (DPSCs) were identified in 2000, which have a self-renewal ability, high proliferative ability, and multilineage differentiation potential such as adipogenic, neurogenic, and osteogenic lineages [5,6,7]. DPSCs are considered to be a type of mesenchymal stem cell (MSC) and have higher clonogenic and proliferative abilities than bone marrow stem cells (BMSCs). Moreover, DPSCs can be easily collected from extracted teeth such as wisdom teeth; this is less invasive than collecting BMSCs and has no ethical issues. Therefore, DPSCs are a promising cell source for regenerative medicine.

Cell sheet therapy is used for regenerative medicine of the heart, liver, cornea, and periodontal tissue [8,9,10,11]. In fracture healing, cell sheet therapy enables more direct application of DPSCs to the fracture site than cell injection, in which cells migrate to various tissues. Therefore, cell sheet therapy effectively promotes fracture healing [12]. Cell sheet technology makes it easy to harvest stem cells without enzymes or scaffolds by a temperature response, and enables transplantation of live cells with minimal damage [13]. Furthermore, cell therapy in regenerative medicine requires safe, inexpensive, and highly efficient culture conditions. Several studies have used gene transfer and growth factors to promote efficient osteogenesis. Recombinant bone morphogenic protein (BMP) has been reported to induce osteogenesis [14]. However, recombinant proteins are expensive, unstable, and involved in the development of cancer [15]. In contrast, several studies have reported that small chemical compounds induce bone formation. In our previous study, we reported a highly efficient method to induce osteogenic differentiation using DPSCs sheets treated with 4-(4-methoxyphenyl)pyrido[40,30:4,5]thieno[2,3-b]pyridine-2-carboxamide (TH), a small molecule derivative of helioxanthin, in a mouse model [16]. However, the mechanism of fracture healing by the transplanted DPSCs remains unknown. Moreover, the localization of the DPSCs after transplantation and their involvement in fracture healing remain unclear.

Therefore, in the present study, we investigated fracture healing using the established culture method of DPSCs. Furthermore, we analyzed the localization of the DPSCs after their transplantation.

## 2. Results

### 2.1. Osteogenic Differentiation of DPSCs Treated with TH in Short-Term Culture

**Figure 1:**
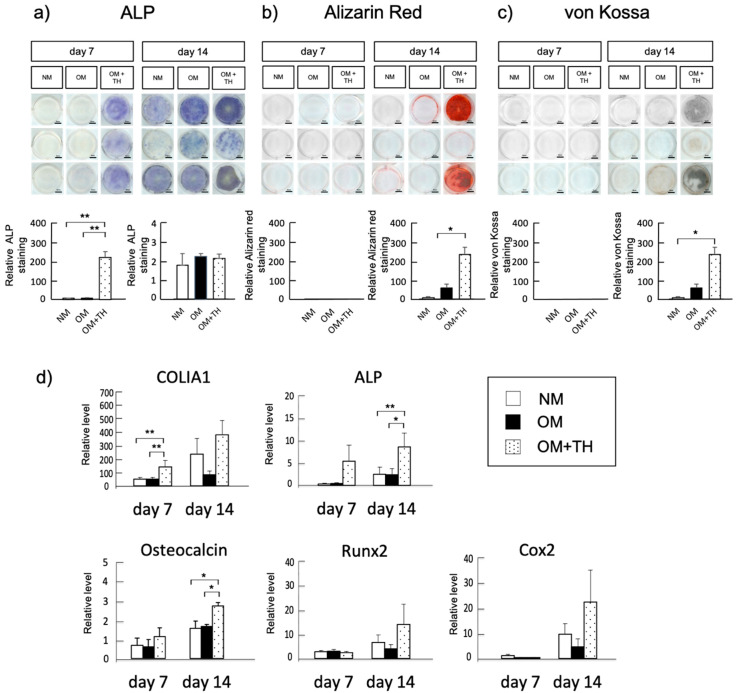
Osteogenic differentiation of dental pulp stem cells (DPSCs) treated with 4-(4-methoxyphenyl)pyrido[40,30:4,5]thieno[2,3-b]pyridine-2-carboxamide (TH) in short-term culture DPSCs were cultured in normal medium (NM) or osteogenic medium (OM) with or without TH for 7 or 14 days. (**a**) cells were stained for alkaline phosphatase (ALP) to detect ALP activity (*n* = 3), and the staining was quantified in arbitrary units. Scale bar is 5 mm. (**b**) cells were stained with Alizarin Red to detect mineralization (*n* = 3), and the staining was quantified in arbitrary units. (**c**) cells were subjected to von Kossa staining to detect mineralization (*n* = 3), and the staining was quantified in arbitrary units. (**d**) real-time PCR to determine the expression levels of osteogenic differentiation markers (*n* = 3). Error bars represent the standard error of the mean (SEM). Statistical analyses were performed using one-way ANOVA (* *p* < 0.05, ** *p* < 0.01). All experiments were repeated three times.

We analyzed the effect of TH on osteogenic differentiation of DPSCs in short-term culture. Alkaline phosphatase (ALP) staining showed that ALP activity of DPSCs cultured in osteogenic medium (OM) with TH was higher than that of DPSCs cultured in normal medium (NM) or OM at days 7 (Figure 1a). Moreover, Alizarin Red and von Kossa staining showed that DPSCs cultured in OM with TH had higher calcification than DPSCs cultured in NM and OM at day 14 (Figure 1b,c). Real-time PCR indicated that OM+TH-treated DPSCs have significantly higher expression levels of type I collagen alpha 1 (COLIA1) on day 7, and ALP and osteocalcin on day 14 than NM and OM-treated DPSCs. In contrast, no statistically significant differences were found in the expression levels of Runx2, a transcription factor involved in the differentiation of osteoblasts and chondrocytes, or Cox2, an enzyme regulating prostaglandins, at 14 days in the three groups (Figure 1d). These data confirmed the results of ALP, von Kossa, and Alizarin staining and indicated that OM+TH-treated DPSCs promote osteogenic differentiation more efficiently than the conventional osteogenic culture method.

### 2.2. Fracture Healing by Transplantation of OM+TH-Treated DPSCs In Vivo

**Figure 2:**
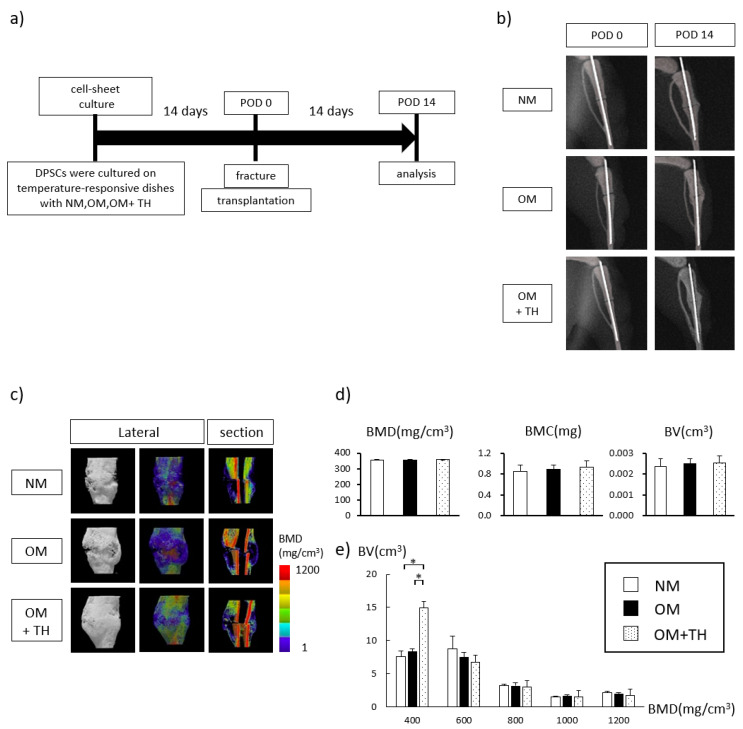
Radiological findings of callus formation induced by the transplantation of DPSCs into mice 14 days after surgery; (**a**) this is experimental procedure. DPSCs sheets were cultured in NM, OM, or OM+TH. Mice underwent the tibia fracture operation. DPSCs sheets were transplanted in tibiae fracture sites. On POD 14, the fractured tibiae were harvested. (**b**) X-ray images of the fracture sites of mice at POD0 and PO14. (**c**) representative three-dimensional (3D) micro-CT images of fractured tibiae transplanted with DPSCs cultured in NM or OM with or without TH (*n* = 9). Lateral views and sagittal images are shown. Color-mapped images were created from bone mineral density (BMD) values. (**d**) quantification of BMD, bone mineral content (BMC), and bone volume (BV) of a 5 by 5 by 4-mm cuboid from the callus in which the fracture line was adjusted to be at the center. (**e**) BMD was calculated in every 200 mg/cm^3^ region and the BV values of each BMD region are shown. Data are shown as means ± SEM of seven mice in each group. * *p* < 0.05 by one-way ANOVA.

To examine fracture healing by the transplantation of OM+TH-treated DPSCs, we cultured DPSCs in 12-well temperature-responsive dishes (3.5 cm^2^/well) with NM, OM, or OM+TH for 14 days, and then transplanted them into left tibia fracture sites. Fourteen days after the transplantation, we harvested the fractured tibiae (Figure 2a). Images of X-ray were taken immediately after surgery (POD 0) and 14 days after surgery (POD 14) to observe changes in the fractured region (Figure 2b). In terms of gross appearance, three-dimensional (3D) micro-CT showed that OM+TH-treated DPSCs transplanted into fractured tibiae tended to form larger calluses than the other DPSCs (Figure 2c). We analyzed callus formation around the fracture line. Figure 2c shows representative images of bone mineral density (BMD) quantification in the callus visualized by color codes constructed by 3D-CT data. Quantitative analysis revealed that BMD, bone mineral content (BMC), and bone volume (BV) were not significantly different among all groups (Figure 2b). However, in accordance with a previous report, the callus area was defined as regions with BMD values of 200–700 mg/cm^3^ [17]. Therefore, we subdivided BMD and BV. As a result, OM+TH-treated DPSCs exhibited a significantly increased BV in the BMD 400 mg/cm^3^ region compared with the other type of DPSCs (Figure 2e).

**Figure 3:**
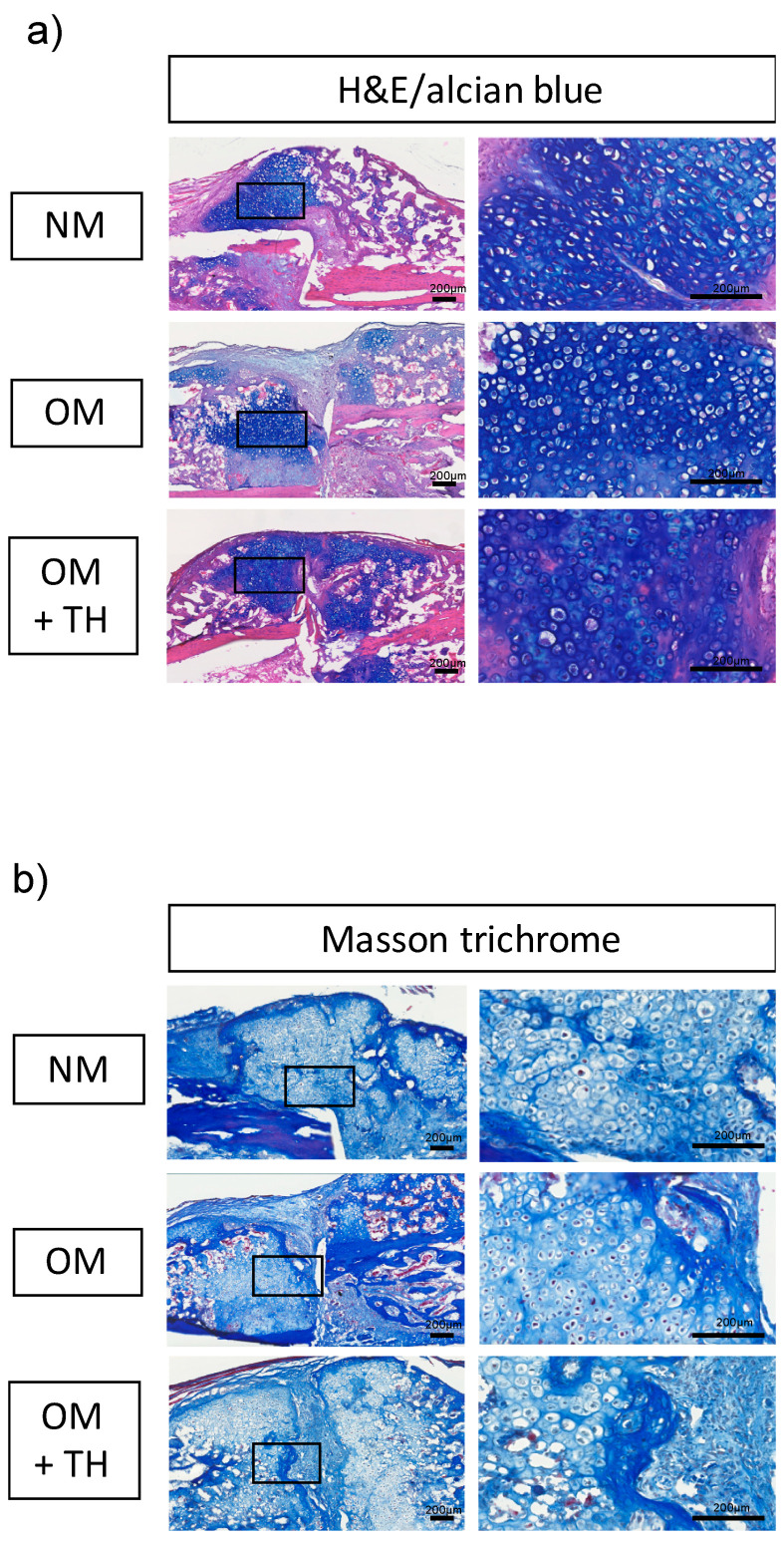
Histological findings of callus induced by the transplantation of DPSCs; histological findings of fractured tibiae transplanted with DPSCs cultured in NM or OM with or without TH. (**a**) representative images of hematoxylin and eosin (H&E) and alcian blue double staining of callus sections of mouse fractured tibiae 14 days after transplantation of DPSCs cultured in NM or OM with or without TH (10^−6^ M). Bars, 200 μm. (**b**) representative images of Masson’s trichrome staining of callus sections of mouse fractured tibiae 14 days after transplantation of DPSCs cultured in NM or OM with or without TH (1 × 10^−6^ M). Bars, 200 μm. High-magnification images of the outlined areas in the left photographs of A and B are shown in the right photographs.

We performed histological analyses to investigate bone regeneration in endochondral ossification. Hematoxylin and eosin (H&E) and alcian blue double staining showed that the area of alcian blue-positive cartilaginous tissue was larger for OM+TH-treated DPSCs compared with the other groups (Figure 3a). Furthermore, the cell morphology of the calluses was similar in all groups (Figure 3a). We performed Masson’s trichrome staining to visualize calcification of new bone formation from cartilage. Masson’s trichrome staining demonstrated that OM+TH-treated DPSCs facilitated more bone mineralization than the others and more efficiently treated the fracture line, which was stained in pale blue (Figure 3b). Thus, OM+TH-treated DPSCs promoted cartilaginous callus formation.

### 2.3. Functions of Transplanted DPSCs

**Figure 4:**
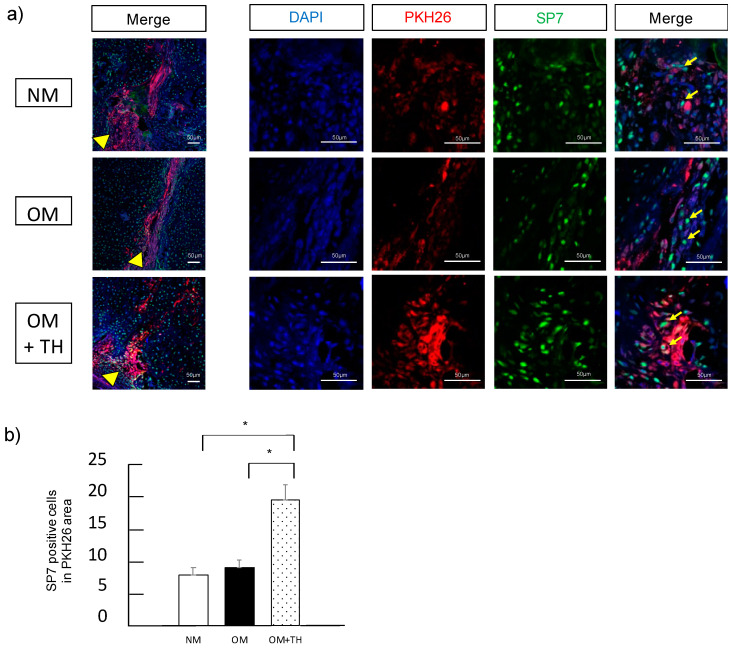
Immunofluorescence analysis of callus induced by transplantation of DPSCs; PKH26-labeled DPSCs were cultured in NM, OM, or OM with TH before transplantation. (**a**) fractured tibiae were stained with SP7 after DPSC transplantation. Nuclei were visualized by DAPI in blue. PKH26 is shown in red and SP7 is shown in green. The arrowheads indicate the fracture line. The arrows indicate cells that are positive for both PKH26 and SP7. (**b**) quantification of SP7-positive cells. Data are shown as means ± SEM. * *p* < 0.05 by one-way ANOVA. Bars, 50 μm.

To investigate whether transplanted DPSCs were localized in the fracture site and involved in fracture healing, we performed fluorescence histological staining using PKH26, a low toxicity red fluorescent label for live cells and immunofluorescence staining using SP7, a marker of mature osteoblasts. We found that transplanted DPSCs stained with PKH26 were localized in the fracture site. Moreover, transplanted DPSCs showed SP7 expression (Figure 4a). Analysis of SP7-positive cells in the area of PKH26 expression revealed that OM+TH-treated DPSCs had significantly higher SP7 expression than the other groups (Figure 4b). These results suggested that OM+TH-treated DPSCs were localized in the fracture site and were involved in fracture healing.

## 3. Discussion

In the present study, we examined the localization of DPSCs after their transplantation in a mouse fracture model. Mesenchymal stem cells can be harvested from bone marrow, fat, skin, and dental pulp. Collecting bone marrow mesenchymal stem cells and adipose stem cells is invasive to the body because they are harvested from bone marrow or subcutaneous fat. In contrast, DPSCs are easily collected from extracted teeth. DPSCs have higher clonogenic and proliferative potentials than BMSCs [6]. Therefore, DPSCs are considered to be an important and accessible cell source for regenerative medicine. However, the volume of DPSCs which we obtain by dental pulp is limited. Furthermore, the amount of dental pulp tissue decreases with age owing to the formation of secondary dentin and narrowing of the dental pulp cavity [18]. To apply DPSCs clinically, it is necessary to secure a large number of cells. Several studies have reported the development of scaffolds [19] and the use of growth factors [14] and compounds [20], to enable highly efficient cell proliferation and induction of bone differentiation. In mammals, osseous tissues are formed by two processes: Membranous and endochondral ossifications during embryogenesis. Membranous bone formation generates numerous craniofacial bones directly from mesenchymal condensations. In contrast, endochondral ossification, the main process that forms most of the mammalian skeleton, is formed by cartilage intermediates [21]. In a previous study, we established a highly efficient method to induce osteogenic differentiation using DPSC sheets treated with TH, which were transplanted into a mouse calvarial defect model [16]. However, the localization of the DPSCs after transplantation remained unknown. In the present study, we investigated the localization of the DPSCs after transplantation, and their involvement in fracture healing in a mouse fracture model.

Xanthine derivatives are organic compounds in tissues and body fluids [22]. TH was found to induce bone formation activity by screening compound libraries [23]. Moreover, TH has been reported to possess BMP-dependent osteogenic activity in mouse osteoblast cell line MC3T3-E1 and promote bone formation in a cranial bone defect mouse model [23,24]. Although the mechanism of TH promoting osteogenic differentiation remains unknown, Amano et al. reported that TH inhibits the hydrolase activity of phosphodiesterase for cyclic guanosine monophosphate by promoting nitric oxide production and promotes bone formation by suppressing osteoclast differentiation [25]. In a previous study, the optimal concentration of TH for the most effective osteogenic differentiation of DPSCs was approximately 1 × 10^−6^ M, and OM was required for osteogenic induction when DPSCs were cultured with TH [16]. Similarly, we demonstrated that OM+TH-treated DPSCs promoted osteogenic differentiation compared with NM and OM by ALP, Alizarin, and von Kossa staining. Moreover, real-time PCR showed that OM+TH-treated DPSCs had significantly higher ColIa1, ALP, and osteocalcin expression levels than NM and OM-induced DPSCs. In addition, OM+TH-treated DPSCs on day 14 showed increased COX2 expression levels. Therefore, we suggested that COX2 upregulated prostaglandin E_2_ production and promoted osteogenic differentiation [26]. Taken together, we consider that TH treatment will shorten the culture period of DPSCs and hence contribute to cost reduction.

Fracture healing has two processes after the inflammatory response: Membranous and endochondral ossifications. Membranous ossification occurs when mesenchymal stem cells differentiate directly into osteogenic osteoblasts. However, endochondral ossification is a process mediated by cartilage intermediates before bone is formed by osteoblasts [27]. In this study, the rate of differentiation of OM+TH-treated DPSCs into osteoblasts was not significantly different from the other groups. However, OM+TH-treated DPSCs had significantly increased BV in the BMD 400 mg/cm^3^ region compared with the other groups, which is the callus area. Moreover, a large amount of cartilaginous tissue was confirmed upon H&E and alcian blue double staining, which was consistent with the results of micro-CT analysis of the callus.

Fracture healing is complicated and regulated by various factors such as MSCs, BMP, parathyroid hormone, and Hedgehog. Accumulation of MSCs in fracture healing is regulated by cytokines released by CXCL12, chemokines associated with the induction of MSCs, also known as stromal cell-derived factor 1 [28]. MSCs are a major cellular contributor to fracture healing. In previous studies, intravenous administration of human or mouse BMSCs contributed to fracture healing [29,30]. BMSCs circulate throughout the body after intravenous administration, the administered BMSCs are mostly retained in the lungs, and the homing rate to fractures is 1–2%. In this study, we demonstrated that DPSCs fluorescently stained with PKH26 and transplanted were localized in the fracture site [31,32]. Moreover, transplanted DPSCs stained with PKH26 showed mature osteoblasts by SP7 expression. Therefore, we suggested that transplanted DPSCs were not only directly involved in fracture healing but also released some cytokines that recruit endogenous MSCs, and OM+TH-treated DPSCs promoted bone regeneration [28]. The transplantation of OM+TH-treated DPSC sheets into the fracture sites maximized the effect of fracture healing by DPSCs.

We have reported that the OM+TH-treated DPSC sheet method is a simple and safe transplantation method [16]. Bone regenerative therapy, using transplantation of DPSC sheets in combination with TH, requires no scaffolds or growth factors, thereby avoiding the problems associated with invasive autologous bone harvesting and the ethical issues associated with allogenic bone transplantation and recombinant protein use. Moreover, previous studies have reported that DPSC sheets have higher immunosuppressive activity and ability to suppress T cell responses than BMSCs [33,34]. These results indicate that DPSC sheets are clinically useful. In a previous study, we reported bone regeneration using OM+TH-treated DPSC sheets. In the present study, we demonstrated that the transplanted DPSCs were localized in the fracture site and directly affected fracture healing. We considered that this study was able to obtain new knowledge of cellular kinetics of the transplanted DPSCs in fracture healing.

There are also some limitations to this study. We demonstrated transplanted DPSCs were localized on fracture sites and directly involved in fracture healing Moreover, transplanted OM+TH-treated DPSCs promoted bone regeneration. However, to clarify whether transplanted OM+TH-treated DPSCs release cytokines, we should analyze mechanism of transplanted DPSCs in the fracture healing. In addition, to apply the OH+TH-treated DPSCs sheets in a clinical setting, it is necessary that the OH+TH-treated DPSCs sheets can be stored for a long time and immediately used without reducing effect for clinical application of OH+TH-treated DPSCs sheets.

## 4. Materials and Methods

### 4.1. Cell Isolation and Culture

The study was performed after all patients had provided written informed consent, which was approved by the institutional ethics committee of the Faculty of Medicine, Tokyo Medical University, Japan (approval No. T2019-0142, approved on 2 November 2019). DPSCs were collected from dental pulp of wisdom teeth from six healthy patients (18–29 years old) at Tokyo Medical University Hospital. The dental pulp was digested in 3 mg/mL collagenase type I (Sigma-Aldrich, Darmstadt, Germany) for 45 min at 37 °C. Single cell suspensions were obtained by passing the cells through a 70-μm strainer. The cells were seeded on 100-mm dishes at 1 × 10^5^ cells each and cultured in alpha minimum essential medium (αMEM; Gibco/BRL, Cheshire, UK) supplemented with 15% fetal bovine serum (FBS; Biowest, Nuaillé, France) and 1% penicillin-streptomycin-amphotericin B suspension (PSA; WAKO Pure Chemical Industries, Osaka, Japan). Cells were passaged at 70% confluence. DPSCs between passage 3 and 5 were used in all experiments. DPSCs were transferred to 12-well plates (BD, Franklin Lakes, NJ, USA) and an Upcell (Cell Seed, Tokyo, Japan), and cultured in normal medium (NM) (αMEM supplemented with 10% FBS and 1% PSA), OM (αMEM supplemented with 10% FBS, 1% PSA, 10 nM dexamethasone (Dex; Wako Pure Chemical Industries, Osaka, Japan), 10 mM β- glycerophosphate (β-GP; Sigma-Aldrich, Darmstadt, Germany), and 100 μM L-ascorbate-2-phosphate (AsAp; Wako Pure Chemical Industries, Osaka, Japan)), or osteogenic medium with TH (Tokyo Chemical Industry, Tokyo, Japan) at the optimal concentration of 1 × 10^−6^ M as described previously [16].

### 4.2. Alkaline Phosphatase Staining

DPSCs were cultured in NM or OM with or without TH for 14 days. ALP staining was performed as described previously [35]. Briefly, the cells were washed with phosphate-buffered saline (PBS), fixed in 70% ethanol, and stained for 10 min with 0.01% naphthol AS-MX phosphate (Sigma-Aldrich, Darmstadt, Germany) using 1% N,N-dimethyl formamide (Wako Pure Chemical Industries, Osaka, Japan) as the substrate and 0.06% Fast BB salt (Sigma-Aldrich, Darmstadt, Germany) as a coupler. Quantification of relative staining intensities was calculated by ImageJ software ver.1.53. (National Institutes of Health, Bethesda, MD, USA).

### 4.3. Alizarin Red S Staining

DPSCs were washed with Ca^2+^-free PBS and fixed in 10% formaldehyde in PBS for 10 min at 4 °C. After washing with distilled water, the cells were stained in an Alizarin Red S (Wako Pure Chemical Industries, Osaka, Japan) solution for 15 min at room temperature. Quantification of relative staining intensities was calculated by ImageJ software ver.1.53.

### 4.4. Von Kossa Staining

DPSCs were washed with PBS and fixed in 100% ethanol for 10 min. After washing with distilled water, the cells were soaked in a 5% silver nitrate solution and then exposed to ultraviolet light for 30 min, after which the cells were washed with distilled water. A 5% thiosulfate solution was applied for 3 min and then the cells were washed in water. Quantification of relative staining intensities was calculated by ImageJ software ver.1.53.

### 4.5. Real-Time Reverse Transcription-Polymerase Chain Reaction Analysis

Total RNA was isolated from DPSCs cultured in NM or OM with or without TH using ISOGEN (Invitrogen, Carlsbad, MA, USA). Reverse transcription was performed using a QuantiTect Reverse Transcription kit (Qiagen, Venlo, Netherland) in accordance with the manufacturer’s instructions. Real-time polymerase chain reaction was performed in a Light Cycler 96 (Roche Diagnostics, Basel, Switizerland) using THUNDERBIRD SYBR qPCR Mix (Toyobo, Osaka, Japan) under the following conditions: 95 °C for 60 s and then 45 cycles at 95 °C for 10 s, 65 °C for 30 s, and 72 °C for 45 s. Real-time polymerase chain reaction was conducted to analyze *ALP* and *COLIA1*, early markers of osteoblast differentiation, *osteocalcin*, a mature osteoblast marker, *Runx2*, an osteoprogenitor marker, and *COX2*, a marker enzyme regulating prostaglandins. *GAPDH* was used as an endogenous control. All reactions were run in triplicate. The primer sequences are presented in Table 1.

### 4.6. Transplantation of a DPSC Sheet into a Mouse Tibia Fracture Model

Animal experiments were performed in accordance with a protocol approved by the Animal Care and Use Committee of the Faculty of Medicine, Tokyo Medical University.

Eight-week-old NOD.CB17-Prkdc^scid^/J (NOD SCID) male mice (CLEA Japan, Tokyo, Japan) were employed for the fracture model. Nine mice were used for each group. Each mouse was anesthetized with medetomidine (0.75 mg/kg), midazolam (4.0 mg/kg), and butorphanol tartrate (5.0 mg/mL) via intraperitoneal injection. A skin incision was made in the left tibia and a blunt dissection of the muscle was made to expose the tibia. Osteotomy was performed using a diamond cutting disc at the mid-diaphysis. The bone marrow cavity of the fractured tibia was stabilized by inserting a 23 G spinal needle (TOP, Tokyo, Japan) [36,37]. DPSC sheets were cultured on temperature-responsive dishes in NM or OM with or without TH for 14 days. Then, the DPSCs were stained with PKH26 (Sigma-Aldrich, Darmstadt, Germany) in accordance with the manufacturer’s protocol. DPSC sheets were removed at room temperature for 30 min, and then transplanted so that they were wrapped around the tibia like a bandage on the fracture line. The incision was closed with 6-0 nylon sutures, and radiography was conducted to confirm the fracture site. After mice were euthanized on day 14, the fractured tibiae were harvested.

### 4.7. Radiological Analysis

X-ray photographs of the left tibiae of six mice from each group were taken using a portable dental X-ray unit (PORT-X III, MORITA, Osaka, Japan). Micro-CT scanning was performed using a microfocus X-ray CT system (SMX-90CT, Shimadzu, Kyoto, Japan) under the following conditions: Tube voltage, 90 kV; tube current, 110 mA; field of view (XY), 13.5 mm. The resolution of one CT slice was 512 by 512 pixels. The three-dimensional construction software package TRI/3D-BON (Ratoc System Engineering, Tokyo, Japan) was used for bone morphometric analysis. The images of bone and callus were constructed in a 5 by 5 by 4-mm cuboid at the callus where a fracture line was adjusted to be the center. BMC, BMD, and BV for each sample were calculated.

### 4.8. Histological Analysis

The fractured tibiae were fixed in 4% PFA overnight and decalcified 10% ethylenediaminetetraacetic acid solution for 21 days. The decalcified samples were embedded in OCT compound (Sakura Finetek Japan, Tokyo, Japan) for cryosectioning. Frozen samples were sectioned at a thickness of 10 μm using a cryostat (Cryostat Microm HM500; Thermo Fisher Scientific, MA, USA). The frozen sections were stained with H&E and alcian blue, or Masson’s trichrome in accordance with the standard protocol [38]. Images were obtained using a slide scanner (Nano Zoomer XR; HAMAMATSU, Shizuoka, Japan).

### 4.9. Immunofluorescence Staining

To investigate transplanted DPSCs were localized and turned into new bone in the fracture site, we performed immunofluorescence staining. Frozen sections of fractured tibiae were washed with PBS twice for 5 min and fixed with 4% paraformaldehyde for 30 min. Before blocking, sections were heated into Tris-EDTA solution for 30 min at 110 °C for antigen retrieval. After cooling down to room temperature for 30 min, these sections were blocked by 10% normal goat serum (NGS) in PBS with 0.1% Teen-20 (PBT) for 1 h, then incubated with antibodies against SP7 (1:500; ab209484, Abcam, Cambridge, UK) overnight at 4 °C. Sections were washed with PBS twice for 5 min, then incubated with the goat anti-human IgG Alexa Fluor 488 (1:400; Invitrogen) for 60 min at room temperature. Nuclear staining was performed by ProLong™ Glass Antifade Mountant with NucBlue™ Stain. (Thermo Fisher Scientific, Waltham, MA, USA) Fluorescence images were captured by LSM700 microscope (ZEISS, Oberkochen, Germany). We quantified the number of the SP7 immunostaining. The number of SP7 positive cells was calculated by the number of SP7 positive cells per PKH26 area (1000 μm^2^) using ImageJ software ver.1.53.

### 4.10. Statistical Analysis

Data are expressed as means ± SEM. Significant differences were evaluated by one-way ANOVA for comparison using SPSS 27.0 software (IBM, Armonk, NY, USA). *p* < 0.05 was considered as significant.

## 5. Conclusions

We found that OM+TH-treated DPSCs promoted fracture healing. Moreover, transplanted DPSCs had localized to the fracture site and were directly involved in fracture healing. Thus, transplantation of OM+TH-treated DPSCs may be useful for fracture healing in terms of safety and convenience.

## Figures and Tables

**Table 1:** Sequence information of primers used for quantitative real-time PCR.

Gene	Primer Sequences (Forward and Reverse, 5′-3′)	Accession
*GAPDH*	GAAGGTGAAGGTCGGAGTCA	BC023632
	GAAGATGGTGATGGGATTTC	
*COLIA1*	GTGCTAAAGGTGCCAATGGT	NM_000088
	CTCCTCGCTTTCCTTCCTCT	
*ALP*	ATGAAGGAAAAGCCAAGCAG	NM_000478
	ATGGAGACATTCTCTCGTTC	
*Osteocalcin*	GGCAGCGAGGTAGTGAAGAG	NM_199173
	AGCAGAGCGACACCCTAGAC	
*Runx2*	CAGACCAGCAGCACTCCATA	NM_004348
	CAGCGTCAACACCATCATTC	
*Cox2*	CCTCCTGTGCCTGATGATTGC	M90100
	TGGCCCTCGCTTATGATCTG	

## Abbreviations

ALP	alkaline phosphatase
BMC	bone mineral content
BMD	bone mineral density
BMP	bone morphogenic protein
BMSCs	bone marrow stem cells
BV	Bone volume
COLIA1	Type I collagen alpha 1
DPSCs	human dental pulp stem cells
FBS	fetal bovine serum
MSCs	mesenchymal stem cells
NM	normal medium
OM	osteogenic medium
PSA	penicillin/streptomycin/amphotericin B
PBS	phosphate-buffered saline
SEM	standard error of the mean
TH	4-(4-methoxyphenyl) pyrido[40,30:4,5]thieno[2,3-b]pyridine-2-carboxamide
αMEM	alpha-modified Eagle’s medium
